# Hepatitis C virus-associated B-cell non-Hodgkin lymphomas: immune evasion, multistep lymphomagenesis, and candidate biomarkers for risk stratification and disease monitoring

**DOI:** 10.3389/fimmu.2026.1890427

**Published:** 2026-08-14

**Authors:** Guido Carloni, Antonio Ponzetto, Monica Rinaldi

**Affiliations:** 1 Department of Bio-Medical Sciences (DSBM), Institute of Traslational Pharmacology (IFT), National Research Council (CNR), Rome, Italy; 2 Department of Medical Sciences, University of Turin, Turin, Italy

**Keywords:** B-cell non-Hodgkin lymphoma, biomarkers, DAAs, hepatitis C virus, immune evasion, mixed cryoglobulinemia, multistep lymphomagenesis, viral persistence

## Abstract

**Background:**

Hepatitis C virus (HCV)-associated B-cell non-Hodgkin lymphomas represent a compelling model of virus-driven oncogenesis in which immune evasion and viral persistence sustain chronic antigenic stimulation, B-cell clonal expansion and progressive multistep lymphomagenesis.

**Methods:**

This minireview focuses on mechanisms that connect HCV immune escape with B-cell lymphomagenesis, antiviral treatment responses, and emerging biomarkers for risk stratification and disease monitoring.

**Results:**

HCV persistence is sustained by extensive viral genetic variability (quasispecies formation) and by interference with innate and adaptive immune responses, including RIG-I/TLR3 pathway inhibition, suppression of interferon signaling, dendritic cell and natural killer cell dysfunction, and T-cell exhaustion. These mechanisms are relevant to lymphomagenesis because they maintain a persistent antigenic and inflammatory niche that supports chronic antigenic stimulation, clonal B-cell expansion, cytokine and chemokine dysregulation (e.g., BAFF/BLyS axis), apoptotic escape, and the progressive accumulation of genetic alterations and chromosomal aberrations. Lymphomagenesis thus emerges as a complex process consistent with a multistep model. Antiviral therapy provides a clinical indicator of this model, in which regression of some indolent HCV-associated lymphomas after viral eradication supports virus dependence, whereas incomplete lymphoproliferative response and the need for immunochemotherapy in aggressive lymphomas indicate partial independence from the viral trigger at advanced stages. Emerging biomarkers, including B-cell activation markers, immune-inflammatory mediators, immunogenetic susceptibility factors, microRNAs, and genomic alterations, show promise for risk stratification, and disease monitoring, but require further validation.

**Conclusion:**

Integrating antiviral and antitumor strategies with biomarker-guided patient stratification may improve early cancer diagnosis and treatment prioritization.

## 1 Introduction

Clinical, epidemiological, and experimental evidence has demonstrated the causal association between Hepatitis C virus (HCV) infection and both type II mixed cryoglobulinemia (MCII) and B-cell non-Hodgkin lymphomas (B-cell NHLs) (1–5). MCII is a chronic lymphoproliferative disorder (LPD) prone to progression toward overt B-cell lymphoma (6–9). The overall risk of developing B-cell NHLs in patients with HCV-associated MCII is estimated to be approximately 35-fold higher than in the general population (7). Extensive investigations and epidemiological studies have demonstrated an elevated risk of B-cell NHLs in individuals with chronic HCV infection compared to uninfected individuals (10–12).

HCV contains a positive-sense single-stranded RNA genome of approximately 9,600 nucleotides, replicates through a negative-strand RNA intermediate, and encodes a single polyprotein that is proteolytically processed by viral and cellular proteases into structural (core C, envelope E1 and E2) and non-structural (NS2, NS3, NS4A, NS4B, NS5A, NS5B) proteins (13–15). HCV infection is characterized by circulating viral variants, known as quasispecies, generated by the high mutation rate of the error-prone viral RNA-dependent RNA polymerase (16, 17). These variants facilitate immune escape and may contribute to expanded viral tropism. A genetic basis for HCV lymphotropism has been proposed and may involve both host genetic factors and viral determinants, including the co-stimulatory molecule B7.2 (CD86), viral envelope proteins, and 5′untranslated region (5′UTR) sequences associated with lymphotropic strains. Notably, silencing of the viral sensor retinoic acid-inducible gene 1 (RIG-I), an RNA helicase recognizing the polyuridine motif within the HCV 3′ untranslated region (3′UTR), together with microRNA-122 overexpression, has been shown to favor persistent HCV replication in B cells (18). Immune evasion favors viral persistence, sustains chronic infection, and eventually contributes to carcinogenesis.

This Mini Review describes immune-evasion mechanisms that are directly relevant to viral persistence, chronic HCV infection, B-cell clonal selection, and lymphoma evolution. The same mechanistic framework has been used to discuss the impact of antiviral therapy with direct-acting antiviral agents (DAAs), which target key components of viral replication, and to explore candidate biomarkers as potential tools for identifying patients at risk of developing LPDs during chronic HCV infection. The specific contribution of this Mini Review is to integrate these mechanisms into a pathogenesis-based model depicting a continuum from virus-dependent B-cell stimulation to progressively antigen-independent lymphoma evolution, linking antiviral treatment responses, and candidate biomarkers for risk stratification and disease monitoring. Literature was selected through a PubMED/NCBI search and relevant cited original articles and reviews, prioritizing studies directly addressing HCV-associated B-cell lymphomas, mixed cryoglobulinemia, HCV-immune evasion mechanisms, antiviral treatment outcomes, and candidate biomarkers.

## 2 Immune-evasion mechanisms driving viral persistence

The immune response to HCV involves multiple antiviral pathways. HCV is capable of evading immune clearance through mechanisms that include interference with innate immune signaling pathways, genetic variability, and dysregulation of both innate and adaptive immune responses. The inability of the immune system to completely eliminate HCV ultimately sustains viral persistence and favors progression toward chronic infection.

HCV RNA is detected mainly by RIG-I-like receptors (RLRs) and Toll-like receptors (TLRs). The RNA helicase RIG-I recognizes viral RNA in the cytosol and activates the mitochondrial antiviral-signaling protein (MAVS), a key adaptor in the RIG-I signaling pathway, whereas TLR3 recognizes viral double-stranded RNA replication intermediates in endosomes and activates a key adaptor for TLR3-mediated signaling, the Toll/interleukin-1 receptor (TIR) domain-containing adaptor inducing interferon-β (TRIF). These pathways trigger phosphorylation of interferon regulatory factors IRF3 and IRF7, thereby inducing type I IFN expression (19, 20). Type I IFNs exert their antiviral activity through phosphorylation of the signal transducers and activators of transcription (STAT) STAT1 and STAT2 proteins. These transcription factors associate with IRF9 to form the heterotrimeric complex known as interferon-stimulated gene factor 3 (ISGF3). ISGF3 then translocates into the nucleus, where it binds interferon-stimulated response elements (ISREs) within promoter regions of interferon-stimulated genes (ISGs), thereby promoting transcription of genes with antiviral activity (21–24).

Several viral proteins contribute to suppression of the interferon response. The NS3/4A serine protease interferes with both RIG-I– and TLR3-dependent signaling, thereby reducing endogenous IFN production and the downstream antiviral response (25–27). NS3/4A inhibits RIG-I polyubiquitination, which is required for its activation (28). Early studies demonstrated that NS3/4A cleaves MAVS, the central adaptor protein in the RIG-I pathway (25, 27, 29). Cleavage of MAVS is considered a major mechanism through which HCV suppresses IFN production. NS3/4A also cleaves TRIF, the adaptor molecule of the TLR3 pathway, thereby preventing activation of IRF3 and NF-κB, and ultimately reducing transcription of IFN genes and proinflammatory cytokines such as tumor necrosis factor α (TNF-α) (26, 30).

The HCV Core protein impairs both TLR- and RLR-mediated signaling in dendritic cells, promotes STAT1 inactivation or degradation (31–33), and induces expression of suppressor of cytokine signaling (SOCS) proteins that limit STAT phosphorylation, leading to reduced transcription of antiviral genes (34).

The NS2 protease has been reported to inhibit the functions of TANK-binding kinase 1 (TBK1) and IκB kinase ϵ (IKKϵ), both of which are required for activation of IRF3 and IRF7 and subsequent induction of IFN-β (35), whereas NS5A inhibits IKK activity (36) and interferes with IFN-induced STAT1 phosphorylation (37, 38). Together, these mechanisms reduce the efficiency of viral clearance and allow continued exposure of B cells to viral antigens.

Genetic variability represents a major immune evasion strategy that favors viral persistence. The high replication rate of HCV and the lack of proofreading activity by the viral RNA-dependent RNA polymerase generate heterogeneous viral populations known as quasispecies (39). Variation in the hypervariable region 1 (HVR1) within the E2 envelope glycoprotein reduces the effectiveness of neutralizing antibodies (40–42), and variants arising in the NS3 and NS5 regions can evade HCV-specific CD4^+^ T cell recognition, whereas HCV genomic mutations within CD8^+^ T cell epitopes may interfere with antigen processing, presentation, and recognition (43–45). Thus, genetic variability functions as an immune-evasion mechanism that sustains long-term antigenic stimulation.

Finally, chronic HCV infection is associated with defects in immune cell populations that are essential for the development of effective adaptive immunity. Natural killer (NK) cells may display reduced frequency and cytotoxic activity and may produce IL-10 and transforming growth factor-β (TGF-β), which dampen antiviral responses and impair dendritic cell (DC) function (46, 47).

Viral proteins contribute to this impairment. A recombinant NS5A-derived antigen, corresponding to amino acids 2061–2302 of the HCV polyprotein was reported to inhibit the capacity of plasmacytoid dendritic cells to produce IFN-α (48). The viral Core and E1 proteins have also been shown to interfere with DC maturation, thereby limiting their ability to activate T cells (49). The HCV Core protein can further suppress IL-2 production by DCs, thereby limiting T cell proliferation, and can reduce IL-12 secretion, a cytokine essential for Th1 differentiation (50, 51). DCs from HCV-infected patients may show reduced frequency, decreased expression of the costimulatory molecules CD83 and CD86, and limited capacity to prime T cells (52–54).

Interactions between HCV and dendritic cells may also promote immune tolerance (52, 55). Consistent with this concept, HCV-specific CD8^+^ T cells detected in chronically infected patients frequently exhibit functional exhaustion, characterized by reduced production of IFN-γ, impaired proliferation, defective cytotoxicity, and defective degranulation activity (56–58). Dysfunction affecting both CD4^+^ and CD8^+^ T cell responses is therefore strongly associated with the persistence of HCV infection (59).

Collectively, suppression of immune signaling pathways by several HCV proteins impairs complete viral clearance, favoring chronic infection. The prolonged exposure to HCV antigens maintains an antigenic and inflammatory milieu that, in turn, promotes B-cell stimulation, survival, and clonal selection, thereby creating a permissive environment for lymphomagenesis, as discussed in Section 3.

## 3 Pathogenesis of HCV-associated B-cell NHLs

**Figure 1:**
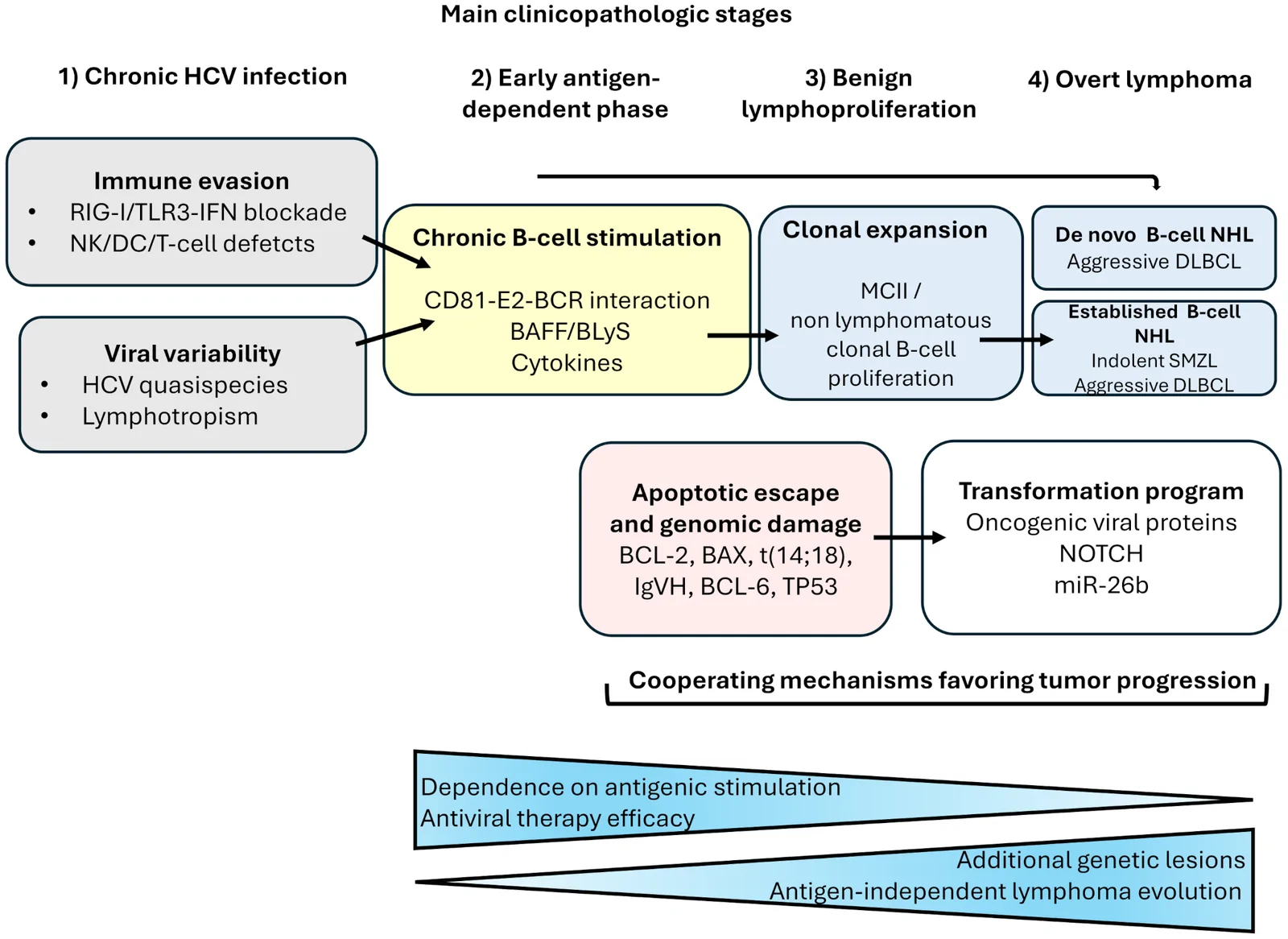
Pathogenesis-based model of HCV-associated B-cell lymphomagenesis. Immune evasion and HCV genetic variability sustain chronic HCV infection and drive chronic B-cell stimulation through CD81-E2-BCR interaction, BLyS/BAFF, and cytokines. This antigen-dependent phase supports clonal expansion and benign lymphoproliferation (MCII/non lymphomatous clonal B-cell proliferation). Cooperating mechanisms – apoptotic escape, genomic damage and transformation programs- promote progression to overt B-cell NHL, including SMZL and DLBCL, or *de novo* neoplastic transformation, including the aggressive DLBCL. Dependence on antigenic stimulation and antiviral therapy efficacy decrease during this progression, whereas additional genetic lesions and antigen-independent lymphoma evolution increase. SMZL, splenic marginal zone lymphomas; DLBCL, diffuse large B-cell lymphoma.

HCV-associated B-cell lymphomagenesis arises from both direct and indirect pathogenic mechanisms that operate within the immune-evasion context described above. **Figure 1** shows a pathogenesis-based model depicting a continuum from virus-dependent B-cell stimulation to progressively antigen-independent lymphoma evolution.

The first step is a prolonged antigen-driven stimulation of B cells by HCV through interaction between the viral E1/E2 envelope proteins and the CD81 receptor expressed on the B-cell surface, with the cooperation of the B-cell receptor (BCR), thereby promoting persistent B-cell proliferation, clonal expansion, and secretion of virus-specific antibodies, particularly in patients with MCII (60). This antigen-dependent phase is supported mainly by evidence from patients with HCV-associated lymphoma, patients with chronic infection or MCII, and studies of primary B cells responsive to HCV antigens.

The preferential use of the *IgV_H_1–69* gene by both HCV-associated B-cell lymphomas and normal B cells in response to E2 viral antigen strengthens the hypothesis that HCV-associated lymphomas derive from clonally expanded B cells stimulated by HCV (61). Conversely, the observation that the BCR expressed by HCV-associated lymphomas rarely recognizes viral proteins suggests that antigen dependence may decline as clones accumulate additional mutations (62).

A second axis involves apoptotic escape and permanent genetic damage (63–65). In HCV-associated B-cell NHLs, chronic B-cell stimulation may coexist with overexpression of the anti-apoptotic *BCL-2* oncogene, increased frequency of t (14, 18) chromosomal translocation, and altered BCL-2/BAX (BCL-associated X) ratio. Furthermore, HCV infection has been associated with increased mutation rate in *IgV_H_*, *BCL-6*, *TP53*, and *β-catenin* genes in primary B-cells, established B-cell lines, and lymphoma samples from HCV-infected patients, consistent with a possible hit-and-run mechanism. This process may explain how an initially antigen-driven lymphoproliferation can become progressively independent of the viral trigger.

A third axis includes direct or indirect oncogenic effects of viral proteins and inflammatory signaling pathways (66, 67). The evidence for the effects of Core, NS3, NS4B, and NS5A proteins derives largely from experimental systems, including established B-cell or lymphoma cell lines, transgenic or viral-protein overexpression experiments, and hepatocyte-derived models; therefore, these findings should be interpreted as potential cooperating mechanisms rather than as direct clinical evidence of B-cell transformation in patients. The Core protein has been shown to promote cell proliferation and to upregulate TP53 family isoform DNp63 transcription, whereas NS3 exhibits oncogenic activity; *in vitro*, Core together with NS3 proteins may upregulate the generation of reactive oxygen and nitrogen species, thereby impairing DNA repair mechanisms, a process associated with high-grade lymphoma *in vivo*. NS5A can protect against TNF-α and TP53-induced apoptosis and promote tumor growth. Core and NS5A act as transcriptional transactivators for several genes. NS4B, through effects on NS5A hyperphosphorylation, IL-8 transactivation, endoplasmic reticulum stress responses, NF-κB activation, and ERK/JNK signaling, may further support STAT3 activation, matrix metalloproteinase-2 (MMP-2) stimulation, and BCL-2 expression (63).

These oncogenic signals are reinforced by mediators of inflammation and B-cell proliferation. Evidence from patients with HCV-associated B-cell NHL and from patients with MCII supports the relevance of inflammatory mediators in this setting. HCV Core protein can induce IL-6 through TLR2, providing a proliferative signal potentially relevant to MCII and B-cell NHL development (67). B-lymphocyte stimulator factor (BLyS), also known as B-cell activating factor (BAFF), typically activates NF-*κ*B, JNK, and ERK pathways that in turn lead to B-cell proliferation. Increased BLyS/BAFF levels have been reported in HCV-related MCII as well as in B-cell NHLs, linking immune activation with B-cell survival (68).

Several cytokines and chemokines (including IL-2, IL-6, IL-10, IL-12, soluble interleukin-2 receptor (sIL-2R), IFN-γ, TNF-α, MIP-1α and MIP-1β, CXCR-3, CXCL10 and CXCL13) may further shape the lymphoproliferative microenvironment (63). Downregulation of miR-26b in HCV-positive splenic marginal zone lymphomas (SMZL), together with recurrent NOTCH pathway mutations in HCV-positive diffuse large B-cell lymphoma (DLBCL), suggests that post-transcriptional and genomic events contribute to transformation and clinical heterogeneity (69, 70). Unlike experimental viral-protein effects, miR-26b dysregulation and NOTCH pathway mutations are supported mainly by observations of lymphoma tissues from patients with HCV-associated lymphomas and may represent later events associated with transformation within the multistep process.

Overall, according to the multistep model of HCV-driven lymphomagenesis, HCV immune evasion sustains viral persistence; persistence maintains chronic antigenic and inflammatory stimulation; these signals promote B-cell expansion and survival; impaired apoptotic control and the progressive accumulation of genetic aberrations eventually allow a subset of clones to evolve toward overt B-cell NHL. Over time, this process may become increasingly independent of the etiologic trigger. Notably, the higher incidence of NHL observed in patients with mixed cryoglobulinemia further supports this model (71). This model explains why some indolent lymphoproliferative disorders regress after antiviral HCV eradication, whereas advanced or aggressive lymphomas may require combined antiviral and chemotherapeutic approaches, as discussed in the following section.

## 4 Direct-acting antiviral agents: viral targets and lymphoma response

Successful regression of splenic lymphoma with villous lymphocytes was first reported following treatment of HCV infection with standard antiviral IFN-α (72). Subsequently, antiviral therapy was shown to induce regression of several HCV-associated indolent/low-grade B-cell NHLs (73–75).

The discovery of direct-acting antiviral agents (DAAs), which target key components of viral replication, has revolutionized the treatment of HCV infection (76). DAAs include inhibitors of nonstructural proteins, nucleoside/nucleotide analogs and non-nucleoside polymerase inhibitors.

NS5A replication complex inhibitors efficiently block both HCV RNA replication and virion assembly/secretion (77). The antiviral activity of a different class of inhibitors is directed against the HCV NS3/4A protease, which plays a crucial role in generating components of the viral RNA replication complex and can cleave two cellular proteins involved in the interferon (IFN) signaling cascade, thereby attenuating the host antiviral response (78). NS5B polymerase can be targeted by nucleotide analog inhibitors that are incorporated by the NS5B polymerase into the growing RNA chain, resulting in premature chain termination (79). One advantage of the nucleoside/nucleotide analog inhibitors is their pan-genotypic effect. They generate replication-defective mutants, thereby providing a high barrier to the emergence of resistant mutants (80).

DAA-based regimens achieved excellent sustained virologic response (SVR) rates, with high viral eradication rates across genotypes (81, 82). However, viral eradication in patients with diverse HCV-associated low-grade/indolent B-cell NHL subtypes did not always translate into complete control of lymphoproliferative disease (81, 83). Antiviral therapy alone is unlikely to be sufficient for aggressive HCV-related NHLs. Aggressive DLBCL generally requires standard immunochemotherapy, with rituximab plus cyclophosphamide, doxorubicin, vincristine, and prednisone (R-CHOP) remaining a standard treatment in current guidelines (84, 85). DLBCL has historically been associated with poor outcome. In a retrospective study, Hosry and colleagues highlighted the negative impact of chronic HCV on survival of DLBCL patients and reported that antiviral therapy was associated with better cancer response to chemotherapy and improved 5-year overall survival (86).

These findings support the concept that lymphoma regression after antiviral therapy may reflect a lymphoproliferative process dependent on HCV-driven antigenic stimulation, whereas partial or absent lymphoproliferative responses despite SVR may indicate progression toward antigen-independent stages of lymphomagenesis, as described in Section 3. These observations provide a rationale for investigating the candidate biomarkers discussed in the following section, which may help identify patients who require closer monitoring and may support timely, histology-driven therapeutic decisions, according to established treatment criteria.

## 5 Candidate biomarkers for risk stratification and disease monitoring

The strong association between HCV infection and B-cell NHLs has prompted the search for molecular signatures that can predict disease progression and clinical outcomes. The development of accessible diagnostic and prognostic tools to detect individuals at higher risk of NHL could improve treatment prioritization.

Putative biomarkers, grouped according to their role in the processes of HCV-associated lymphomagenesis, and classified according to their proposed intended use, are shown in **Table 1**.

**Table 1:** Candidate biomarker signatures for risk stratification and disease monitoring during HCV infection.

Mechanistic category	Candidate biomarkers	Clinical setting	Mechanistic/clinical signal	References
B-cell activation	**^±^** IgG3 **^±^** FLC **^±^** VEGF **^±^** IgG2 **^±^** IgG4 **^≠^** sIL-2Rα **^≠^** γ-globulins **^≠^** sCD27 **^≠^** C4	Type II mixed cryoglobulinemia; Cryoglobulinemic vasculitis	Ongoing antigen-driven B-cell stimulation; identification of patients at risk of or with overt B-cell NHL	(88, 89)
B-cell survival pathways	^& **±**^ BLyS/BAFF **^±^** BCR signaling **^±^** NF-κB	Mixed cryoglobulinemic syndrome; HCV-positive lymphoma	B-cell maturation; B-cell survival signaling; NF-κB activity	(68, 90)
Innate immunity Immunogenetic susceptibility	^*^ KIR2DS3 ^*^ KIR2DL2 ^*^ KIR2DL3 ^*^ KIR3DL1/HLA-Bw6	LPDs; MCII; B-cell NHL	Host immunogenetic background; influencing progression toward MC or NHL	(91)
Innate immunity Immunogenetic profile	^*^ TLR2 ^*^ IL28B polymorphisms	LPDs	Host profile directing chronic HCV toward hepatic or LPD outcomes	(92)
Dysregulated miRNA expression	**^‡^** miR-26b	SMZL MC	Tumor-suppressive miR-26b associated with HCV-positive SMZL and disease progression	(93, 94)
Signaling pathway dysregulation; genomic alterations	**^‡^** NOTCH mutations ^*^ NOTCH4/HLA-II SNPs	HCV-associated DLBCL; HCV-related LPDs	Transformation from indolent SMZL; categorization of patients at increased lymphoma risk	(69, 95)

HCV, hepatitis C virus; B-cell NHL, B-cell non-Hodgkin lymphoma; FLC, free light chains; VEGF, vascular endothelial growth factor; sIL-2Rα, soluble interleukin-2 receptor alpha; sCD27, soluble CD27; BLyS/BAFF, B-lymphocyte stimulator factor/B-cell activating factor; BCR, B-cell receptor; NF-κB, nuclear factor kappa B; LPDs, lymphoproliferative disorders; KIR, killer cell immunoglobulin-like receptor; MCII, type II mixed cryoglobulinemia; HLA-Bw6: human leukocyte antigen Bw6 serological epitope; TLR2, Toll-like receptor 2; IL28B, interleukin 28B; SMZL, splenic marginal zone lymphoma; miR-26b, microRNA-26b; MC, mixed cryoglobulinemia; DLBCL, diffuse large B-cell lymphoma; HLA-II, human leukocyte antigen class II; SNP, single nucleotide polymorphism; NHL, non-Hodgkin lymphoma.

**^±^**mechanistic correlates; **^≠^**diagnostic markers; & treatment response or monitoring markers; *susceptibility or risk markers; **^‡^**prognostic or progression markers.

Patients with HCV-related mixed cryoglobulinemia represent the subgroup at highest risk of developing B-cell NHLs compared with the general population (87). A major objective is to define biomarker signatures that may support risk stratification, diagnostic assessment, and patient monitoring. HCV-infected patients with indolent B-cell lymphomas are at high risk of progression toward aggressive form of B-cell lymphomas (88). Therefore, it would be of paramount importance to recognize these patients, and to start a combined antiviral and antitumor therapy at an early stage. Several candidates have been suggested as biomarkers of pathogenesis and progression from HCV-related mixed cryoglobulinemia to B-cell NHL. Biomarkers of B-cell activation may identify patients with chronic antigenic stimulation. In HCV-positive patients with MCII, serum levels of IgG3 subclass, free light chains (FLC), and vascular endothelial growth factor (VEGF) were significantly increased, where IgG2 and IgG4 subclasses were reduced, suggesting their potential utility as biomarkers of MCII pathogenesis and lymphoproliferative risk (89). In chronic HCV infection with cryoglobulinemic vasculitis, serum levels of sIL-2Rα, γ-globulins, sCD27, and C4 have been proposed as a serum signature for identifying patients with overt B-NHL (88).

A second group of biomarkers is associated with B-cell survival pathways. Several lines of evidence suggest an important role of BLyS/BAFF, a B-cell survival factor and a TNF family member, because it links chronic immune activation with BCR-related survival signaling and NF-κB activation. Molecular signatures in HCV-positive lymphomas revealed a set of genes whose expression is associated with BCR signaling, functionally coupled to BLyS pathways, which in turn was found to be linked to B-cell maturation stages and NF-κB transcription factor (68). Significantly increased BLyS serum levels have also been demonstrated in mixed cryoglobulinemic syndrome, a systemic vasculitis associated with a high risk of developing lymphoma (90). These data suggest that BLyS may represent a marker of immune activation and a potential therapeutic target; however, further investigations are needed.

A third group includes immunogenetic markers that may influence the direction of chronic HCV disease and that are linked to the innate immunity system discussed in Section 2. The susceptibility to HCV-related diseases seems to be related to components of the innate immune system such as killer cell immunoglobulin-like receptor (KIR) genes, and to the genetic diversity of the KIR/HLA combinations. *KIR2DS3*, *KIR2DL2* and *KIR2DL3* genes have been associated with HCV disease progression, mainly toward lymphoproliferative disorders. Specific *KIR3D/HLA* gene combinations may shift progression toward lymphoma rather than liver disease. Furthermore, a pool of specific KIR/HLA interactions is thought to play a key role in determining whether the lymphoproliferative disorders result in MC rather than in NHL cases (91).

Immunogenetic profiles may play an important role in determining the progression of chronic HCV infections toward different diseases. Because TLR2 and IL28B polymorphisms in combination may have a role in directing HCV progression toward hepatic or lymphoproliferative diseases, they may have potential relevance for risk stratification, although clinical utility remains to be validated (92).

A fourth group includes potential mediators of malignant transformation in HCV-associated B-cell non-Hodgkin lymphoma. The tumor suppressive miR-26b was significantly downregulated in HCV-associated versus HCV-negative patients with SMZL (93). These data, observed not only in NHL but also in MC patients, support the relevance of miR-26b modulation as a potential biomarker of disease progression (94). The NOTCH pathway is recurrently mutated in DLBCL associated with HCV infection. A subgroup of DLBCL arising in patients with HCV infection represents the transformation of a previous, clinically unrecognized indolent SMZL. Deregulation of the NOTCH signaling pathway may therefore predict unfavorable clinical outcomes (69). Significant associations have been reported between two single nucleotide polymorphisms (SNPs) near *NOTCH4* (rs2071286) and *HLA class II* (rs9461776) genes and HCV-related LPDs, with an increased risk of NHL development. Thus, the use of these SNPs has been proposed as potential markers to categorize patients with an increased risk for lymphoma development, which may be useful for therapeutic prioritization (95).

Overall, according to the pathogenic processes they represent, B-cell activation markers may identify patients with ongoing antigen-driven stimulation; BLyS and inflammatory mediators may reflect survival and microenvironmental support; immunogenetic markers may identify host susceptibility; tumor-suppressor and signaling pathway dysregulation may indicate progression toward more autonomous transformation. Prospective validation will be required before these biomarkers can be used as screening tools. Clinically, these biomarker groups may provide a framework for risk stratification and treatment prioritization.

## 6 Conclusion

Virus-associated malignancies represent a substantial global disease burden, affecting approximately 1.5 million individuals each year (96–98). HCV is thought to contribute to nearly 11% of virus-related cancers, with around 160,000 new cancer diagnoses reported worldwide in 2018, mainly hepatocellular carcinoma and B-cell non-Hodgkin lymphomas (99).

The introduction of DAAs has profoundly reshaped the clinical management of HCV infection by enabling viral eradication in the vast majority of patients (100). Nevertheless, lymphoproliferative manifestations may persist despite viral clearance. This apparent discrepancy may reflect the fact that HCV eradication removes upstream drivers of antigenic stimulation, but established B-cell clones with autonomous survival, genomic instability, and transformation programs may persist.

Therefore, HCV-associated B-cell lymphomagenesis represents a model of immune-driven oncogenesis at the intersection of chronic viral infection and tumor progression and can be interpreted as a continuum from immune evasion and persistent antigenic stimulation to antigen-independent tumor evolution.

In this setting, defining candidate biomarker signatures capable of stratifying patients according to their risk of developing lymphoproliferative disorders during chronic HCV infection represents a major unmet clinical need in the DAAs era. The availability of validated biomarkers could improve risk stratification and disease monitoring, and refine the classification of HCV-associated lymphoid malignancies, ultimately supporting tailored therapeutic approaches across both indolent and aggressive disease forms. Furthermore, the identification of high-risk subgroups may allow more timely diagnostic assessment and treatment according to established clinical criteria, with potential benefits for both clinical outcomes and healthcare resource optimization. Within this framework, integrating antiviral and antitumor therapies, alongside biomarker-driven patient stratification, may help advance personalized care and improve prognosis in HCV-associated B-cell malignancies.

## References

[1] Marcucci F Mele A . Hepatitis viruses and non-hodgkin lymphoma: epidemiology, mechanisms of tumorigenesis, and therapeutic opportunities. Blood. (2011) 117:1792–8. doi: 10.1182/blood-2010-06-275818 20959600

[2] Su TH Liu CJ Tseng TC Chou SW Liu CH Yang HC . Hepatitis c viral infection increases the risk of lymphoid-neoplasms: a population-based cohort study. Hepatology. (2016) 63:721–30. doi: 10.1002/hep.28387 26662347

[3] Gisbert JP Garcia-Buey L Pajares JM Moreno-Otero R . Prevalence of hepatitis c virus infection in b-cell non-hodgkin's lymphoma: systematic review and meta-analysis. Gastroenterology. (2003) 125:1723–32. doi: 10.1053/j.gastro.2003.09.025 14724825

[4] Nieters A Kallinowski B Brennan P Ott M Maynadié M Benavente Y . Hepatitis c and risk of lymphoma: results of the european multicenter case-control study epilymph. Gastroenterology. (2006) 131:1879–86. doi: 10.1053/j.gastro.2006.09.019 17087949

[5] de Sanjose S Benavente Y Vajdic CM Engels EA Morton LM Bracci PM . Hepatitis c and non-hodgkin lymphoma among 4784 cases and 6269 controls from the international lymphoma epidemiology consortium. Clin Gastroenterol Hepatol. (2008) 6:451–8. doi: 10.1016/j.cgh.2008.02.011 18387498 PMC3962672

[6] Ferri C Caracciolo F Zignego AL La Civita L Monti M Longombardo G . Hepatitis c virus infection in patients with non-hodgkin's lymphoma. Br J Haematol. (1994) 88:392–4. doi: 10.1111/j.1365-2141.1994.tb05036.x 7803287

[7] Monti G Pioltelli P Saccardo F Campanini M Candela M Cavallero G . Incidence and characteristics of non-hodgkin lymphomas in a multicenter case file of patients with hepatitis c virus-related symptomatic mixed cryoglobulinemias. Arch Intern Med. (2005) 165:101–5. doi: 10.1001/archinte.165.1.101 15642884

[8] Pascual M Perrin L Giostra E Schifferli JA . Hepatitis c virus in patients with cryoglobulinemia type ii. J Infect Dis. (1990) 162:569–70. doi: 10.1093/infdis/162.2.569 2115556

[9] Agnello V Chung RT Kaplan LM . A role for hepatitis c virus infection in type ii cryoglobulinemia. N Engl J Med. (1992) 327:1490–5. doi: 10.1056/NEJM199211193272104 1383822

[10] Alkrekshi A Kassem A Park C Tse W . Risk of non-hodgkin's lymphoma in hcv patients in the United States between 2013 and 2020: a population-based study. Clin Lymphoma Myeloma Leuk. (2021) 21:e832–8. doi: 10.1016/j.clml.2021.06.014 34330674

[11] Giordano TP Henderson L Landgren O Chiao EY Kramer JR El-Serag H . Risk of non-hodgkin lymphoma and lymphoproliferative precursor diseases in us veterans with hepatitis c virus. JAMA. (2007) 297:2010–7. doi: 10.1001/jama.297.18.2010 17488966

[12] Schöllkopf C Smedby KE Hjalgrim H Rostgaard K Panum I Vinner L . Hepatitis c infection and risk of Malignant lymphoma. Int J Cancer. (2008) 122:1885–90. doi: 10.1002/ijc.23416 18271005

[13] Lindenbach BD Rice CM . Unravelling hepatitis c virus replication from genome to function. Nature. (2005) 436:933–8. doi: 10.1038/nature04077 16107832

[14] Bode JG Brenndörfer ED Häussinger D . Hepatitis c virus (hcv) employs multiple strategies to subvert the host innate antiviral response. Biol Chem. (2008) 389:1283–98. doi: 10.1515/BC.2008.147 18713016

[15] Manns MP Buti M Gane E Pawlotsky JM Razavi H Terrault N . Hepatitis c virus infection. Nat Rev Dis Primers. (2017) 3:17006. doi: 10.1038/nrdp.2017.6 28252637

[16] Lohmann V Bartenschlager R . On the history of hepatitis c virus cell culture systems. J Med Chem. (2014) 57:1627–42. doi: 10.1021/jm401401n 24164647

[17] Martell M Esteban JI Quer J Genescà J Weiner A Esteban R . Hepatitis c virus (hcv) circulates as a population of different but closely related genomes: quasispecies nature of hcv genome distribution. J Virol. (1992) 66:3225–9. doi: 10.1128/JVI.66.5.3225-3229.1992 1313927 PMC241092

[18] Chen CL Huang JY Wang CH Tahara SM Zhou L Kondo Y . Hepatitis c virus has a genetically determined lymphotropism through co-receptor b7.2. Nat Commun. (2017) 8:13882. doi: 10.1038/ncomms13882 28067225 PMC5227552

[19] Saito T Owen DM Jiang F Marcotrigiano J Gale M Jr . Innate immunity induced by composition-dependent rig-i recognition of hepatitis c virus rna. Nature. (2008) 454:523–7. doi: 10.1038/nature07106 18548002 PMC2856441

[20] Wang N Liang Y Devaraj S Wang J Lemon SM Li K . Toll-like receptor 3 mediates establishment of an antiviral state against hepatitis c virus in hepatoma cells. J Virol. (2009) 83:9824–34. doi: 10.1128/JVI.01125-09 PMC2747996 19625408

[21] Ivashkiv LB Donlin LT . Regulation of type i interferon responses. Nat Rev Immunol. (2014) 14:36–49. doi: 10.1038/nri3581 24362405 PMC4084561

[22] Müller M Laxton C Briscoe J Schindler C Improta T Darnell JE Jr. . Complementation of a mutant cell line: central role of the 91 kda polypeptide of isgf3 in the interferon-alpha and -gamma signal transduction pathways. EMBO J. (1993) 12:4221–8. doi: 10.1002/j.1460-2075.1993.tb06093.x 7693454 PMC413716

[23] Kessler DS Veals SA Fu XY Levy DE . Interferon-alpha regulates nuclear translocation and dna-binding affinity of isgf3, a multimeric transcriptional activator. Genes Dev. (1990) 4:1753–65. doi: 10.1101/gad.4.10.1753 2249773

[24] Stetson DB Medzhitov R . Type i interferons in host defense. Immunity. (2006) 25:373–81. doi: 10.1016/j.immuni.2006.08.007 16979569

[25] Meylan E Curran J Hofmann K Moradpour D Binder M Bartenschlager R . Cardif is an adaptor protein in the rig-i antiviral pathway and is targeted by hepatitis c virus. Nature. (2005) 437:1167–72. doi: 10.1038/nature04193 16177806

[26] Li K Foy E Ferreon JC Nakamura M Ferreon AC Ikeda M . Immune evasion by hepatitis c virus ns3/4a protease-mediated cleavage of the toll-like receptor 3 adaptor protein trif. Proc Natl Acad Sci USA. (2005) 102:2992–7. doi: 10.1073/pnas.0408824102 15710891 PMC548795

[27] Li XD Sun L Seth RB Pineda G Chen ZJ . Hepatitis c virus protease ns3/4a cleaves mitochondrial antiviral signaling protein off the mitochondria to evade innate immunity. Proc Natl Acad Sci USA. (2005) 102:17717–22. doi: 10.1073/pnas.0508531102 16301520 PMC1308909

[28] Oshiumi H Miyashita M Inoue N Okabe M Matsumoto M Seya T . The ubiquitin ligase riplet is essential for rig-i-dependent innate immune responses to rna virus infection. Cell Host Microbe. (2010) 8:496–509. doi: 10.1016/j.chom.2010.11.008 21147464

[29] Lin R Lacoste J Nakhaei P Sun Q Yang L Paz S . Dissociation of a mavs/ips-1/visa/cardif-ikkepsilon molecular complex from the mitochondrial outer membrane by hepatitis c virus ns3-4a proteolytic cleavage. J Virol. (2006) 80:6072–83. doi: 10.1128/JVI.02495-05 PMC1472616 16731946

[30] Ferreon JC Ferreon AC Li K Lemon SM . Molecular determinants of trif proteolysis mediated by the hepatitis c virus ns3/4a protease. J Biol Chem. (2005) 280:20483–92. doi: 10.1074/jbc.M500422200 15767257

[31] Stone AE Mitchell A Brownell J Miklin DJ Golden-Mason L Polyak SJ . Hepatitis c virus core protein inhibits interferon production by a human plasmacytoid dendritic cell line and dysregulates interferon regulatory factor-7 and stat1 protein expression. PloS One. (2014) 9:e95627. doi: 10.1371/journal.pone.0095627 24788809 PMC4006833

[32] Lin W Choe WH Hiasa Y Kamegaya Y Blackard JT Schmidt EV . Hepatitis c virus expression suppresses interferon signaling by degrading stat1. Gastroenterology. (2005) 128:1034–41. doi: 10.1053/j.gastro.2005.02.010 15825084

[33] Lin W Kim SS Yeung E Kamegaya Y Blackard JT Kim KA . Hepatitis c virus core protein blocks interferon signaling by interaction with the stat1 sh2 domain. J Virol. (2006) 80:9226–35. doi: 10.1128/JVI.00459-06 PMC1563912 16940534

[34] Bode JG Ludwig S Ehrhardt C Albrecht U Erhardt A Schaper F . Ifn-alpha antagonistic activity of hcv core protein involves induction of suppressor of cytokine signaling-3. FASEB J. (2003) 17:488–90. doi: 10.1096/fj.02-0664fje 12551851

[35] Kaukinen P Sillanpää M Nousiainen L Melén K Julkunen I . Hepatitis c virus ns2 protease inhibits host cell antiviral response by inhibiting ikk and tbk1 functions. J Med Virol. (2013) 85:71–82. doi: 10.1002/jmv.23449 23096996

[36] Kang SM Park JY Han HJ Song BM Tark D Choi BS . Hepatitis c virus nonstructural protein 5a interacts with immunomodulatory kinase ikk to negatively regulate innate antiviral immunity. Mol Cells. (2022) 45:702–17. doi: 10.14348/molcells.2022.0018 PMC9589372 35993162

[37] Lan KH Lan KL Lee WP Sheu ML Chen MY Lee YL . Hcv ns5a inhibits interferon signaling through suppression of stat1 phosphorylation in hepatocyte-derived cell lines. J Hepatol. (2007) 46:759–67. doi: 10.1016/j.jhep.2006.11.023 17275127

[38] Kumthip K Chusri P Jilg N Zhao L Fusco DN Zhao H . Hepatitis c virus ns5a disrupts stat1 phosphorylation and suppresses type i interferon signaling. J Virol. (2012) 86:8581–91. doi: 10.1128/JVI.00647-12 22674974 PMC3421739

[39] Farci P . New insights into the hcv quasispecies and compartmentalization. Semin Liver Dis. (2011) 31:356–74. doi: 10.1055/s-0031-1297925 22189976

[40] Hiroishi K Ito T Imawari M . Immune responses in hepatitis c virus infection and mechanisms of hepatitis c virus persistence. J Gastroenterol Hepatol. (2008) 23:1473–82. doi: 10.1111/j.1440-1746.2008.05499.x 18761560

[41] Kato N Ootsuyama Y Sekiya H Ohkoshi S Nakazawa T Hijikata M . Genetic drift in hypervariable region 1 of the viral genome in persistent hepatitis c virus infection. J Virol. (1994) 68:4776–84. doi: 10.1128/JVI.68.8.4776-4784.1994 7518526 PMC236417

[42] Weiner AJ Geysen HM Christopherson C Hall JE Mason TJ Saracco G . Evidence for immune selection of hepatitis c virus (hcv) putative envelope glycoprotein variants: potential role in chronic hcv infections. Proc Natl Acad Sci USA. (1992) 89:3468–72. doi: 10.1073/pnas.89.8.3468 1314389 PMC48889

[43] Puig M Mihalik K Tilton JC Williams O Merchlinsky M Connors M . CD4+ immune escape and subsequent t-cell failure following chimpanzee immunization against hepatitis c virus. Hepatology. (2006) 44:736–45. doi: 10.1002/hep.21312 16941702

[44] Meyer-Olson D Shoukry NH Brady KW Kim H Olson DP Hartman K . Limited t cell receptor diversity of hcv-specific t cell responses is associated with ctl escape. J Exp Med. (2004) 200:307–19. doi: 10.1084/jem.20040638 PMC2211982 15289502

[45] Seifert U Liermann H Racanelli V Halenius A Wiese M Wedemeyer H . Hepatitis c virus mutation affects proteasomal epitope processing. J Clin Invest. (2004) 114:250–9. doi: 10.1172/JCI20985 15254592 PMC449747

[46] Jinushi M Takehara T Tatsumi T Kanto T Miyagi T Suzuki T . Negative regulation of nk cell activities by inhibitory receptor cd94/nkg2a leads to altered nk cell-induced modulation of dendritic cell functions in chronic hepatitis c virus infection. J Immunol. (2004) 173:6072–81. doi: 10.4049/jimmunol.173.10.6072 15528343

[47] De Maria A Fogli M Mazza S Basso M Picciotto A Costa P . Increased natural cytotoxicity receptor expression and relevant il-10 production in nk cells from chronically infected viremic hcv patients. Eur J Immunol. (2007) 37:445–55. doi: 10.1002/eji.200635989 17273991

[48] Amjad M Abdel-Haq N Faisal M Kamal M Moudgal V . Decreased interferon-alpha production and impaired regulatory function of plasmacytoid dendritic cells induced by the hepatitis c virus ns5 protein. Microbiol Immunol. (2008) 52:499–507. doi: 10.1111/j.1348-0421.2008.00067.x 18822084

[49] Lasarte JJ Casares N López-Díaz de Cerio A Baixeras E Labarga P García N . Abnormal priming of cd4(+) t cells by dendritic cells expressing hepatitis c virus core and e1 proteins. J Virol. (2002) 76:5062–70. doi: 10.1128/JVI.76.10.5062-5070.2002 11967322 PMC136154

[50] Kittlesen DJ Chianese-Bullock KA Yao ZQ Braciale TJ Hahn YS . Interaction between complement receptor gc1qr and hepatitis c virus core protein inhibits t-lymphocyte proliferation. J Clin Invest. (2000) 106:1239–49. doi: 10.1172/JCI10323 11086025 PMC381434

[51] Waggoner SN Hall CH Hahn YS . HCV core protein interaction with gC1q receptor inhibits Th1 differentiation of CD4+ T cells via suppression of dendritic cell IL-12 production. J Leukoc Biol. (2007) 82:1407–19. doi: 10.1189/jlb.0207090 17881511

[52] Kanto T Inoue M Miyatake H Sato A Sakakibara M Yakushijin T . Reduced numbers and impaired ability of myeloid and plasmacytoid dendritic cells to polarize T helper cells in chronic hepatitis C virus infection. J Infect Dis. (2004) 190:1919–26. doi: 10.1086/425425 15529255

[53] Della Bella S Crosignani A Riva A Presicce P Benetti A Longhi R . Decrease and dysfunction of dendritic cells correlate with impaired hepatitis C virus-specific CD4+ T-cell proliferation in patients with hepatitis C virus infection. Immunology. (2007) 121:283–92. doi: 10.1111/j.1365-2567.2007.02577.x 17462079 PMC2265942

[54] Auffermann-Gretzinger S . Impaired dendritic cell maturation in patients with chronic, but not resolved, hepatitis C virus infection. Blood. (2001) 97:3171–6. doi: 10.1182/blood.V97.10.3171 11342445

[55] Kanto T Inoue M Miyazaki M Itose I Miyatake H Sakakibara M . Impaired function of dendritic cells circulating in patients infected with hepatitis C virus who have persistently normal alanine aminotransferase levels. Intervirology. (2006) 49:58–63. doi: 10.1159/000087263 16166790

[56] Gruener NH Lechner F Jung MC Diepolder H Gerlach T Lauer G . Sustained dysfunction of antiviral CD8+ T lymphocytes after infection with hepatitis C virus. J Virol. (2001) 75:5550–8. doi: 10.1128/JVI.75.12.5550-5558.2001 11356962 PMC114267

[57] Wedemeyer H He XS Nascimbeni M Davis AR Greenberg HB Hoofnagle JH . Impaired effector function of hepatitis C virus-specific CD8+ T cells in chronic hepatitis C virus infection. J Immunol. (2002) 169:3447–58. doi: 10.4049/jimmunol.169.6.3447 12218168

[58] Missale G Cariani E Ferrari C . Role of viral and host factors in HCV persistence: which lesson for therapeutic and preventive strategies? Dig Liver Dis. (2004) 36:703–11. doi: 10.1016/j.dld.2004.05.006 15570998

[59] Gerlach JT Diepolder HM Jung MC Gruener NH Schraut WW Zachoval R . Recurrence of hepatitis C virus after loss of virus-specific CD4(+) T-cell response in acute hepatitis C. Gastroenterology. (1999) 117:933–41. doi: 10.1016/s0016-5085(99)70353-7 10500077

[60] Quinn ER Chan CH Hadlock KG Foung SK Flint M Levy S . The B-cell receptor of a hepatitis C virus (HCV)-associated non-Hodgkin lymphoma binds the viral E2 envelope protein, implicating HCV in lymphomagenesis. Blood. (2001) 98:3745–9. doi: 10.1182/blood.v98.13.3745 11739181

[61] Chan CH Hadlock KG Foung SK Levy S . V(H)1-69 gene is preferentially used by hepatitis C virus-associated B-cell lymphomas and by normal B cells responding to the E2 viral antigen. Blood. (2001) 97:1023–6. doi: 10.1182/blood.v97.4.1023 11159532

[62] Ng PP Kuo CC Wang S Einav S Arcaini L Paulli M . B-cell receptors expressed by lymphomas of hepatitis C virus (HCV)-infected patients rarely react with the viral proteins. Blood. (2014) 123:1512–5. doi: 10.1182/blood-2013-10-532895 24449209 PMC3945861

[63] Carloni G Fioretti D Rinaldi M Ponzetto A . Heterogeneity and coexistence of oncogenic mechanisms involved in HCV-associated B-cell lymphomas. Crit Rev Oncol Hematol. (2019) 138:156–71. doi: 10.1016/j.critrevonc.2019.04.005 31092372

[64] Vannata B Arcaini L Zucca E . Hepatitis C virus-associated B-cell non-Hodgkin’s lymphomas: what do we know? Ther Adv Hematol. (2016) 7:94–107. doi: 10.1177/2040620715623924 27054025 PMC4802504

[65] Machida K Cheng KT Sung VM Shimodaira S Lindsay KL Levine AM . Hepatitis C virus induces a mutator phenotype: enhanced mutations of immunoglobulin and proto-oncogenes. Proc Natl Acad Sci USA. (2004) 101:4262–7. doi: 10.1073/pnas.0303971101 14999097 PMC384729

[66] Levrero M . Viral hepatitis and liver cancer: the case of hepatitis C. Oncogene. (2006) 25:3834–47. doi: 10.1038/sj.onc.1209562 16799625

[67] Feldmann G Nischalke HD Nattermann J Banas B Berg T Teschendorf C . Induction of interleukin-6 by hepatitis C virus core protein in hepatitis C-associated mixed cryoglobulinemia and B-cell non-Hodgkin’s lymphoma. Clin Cancer Res. (2006) 12:4491–8. doi: 10.1158/1078-0432.CCR-06-0154 16899594

[68] De Re V Caggiari L Garziera M De Zorzi M Repetto O . Molecular signature in HCV-positive lymphomas. Clin Dev Immunol. (2012) 2012:623465. doi: 10.1155/2012/623465 22952554 PMC3431075

[69] Arcaini L Rossi D Lucioni M Nicola M Bruscaggin A Fiaccadori V . The NOTCH pathway is recurrently mutated in diffuse large B-cell lymphoma associated with hepatitis C virus infection. Haematologica. (2015) 100:246–52. doi: 10.3324/haematol.2014.116855 25381127 PMC4803124

[70] Peveling-Oberhag J Arcaini L Hansmann ML Zeuzem S . Hepatitis C-associated B-cell non-Hodgkin lymphomas. Epidemiology, molecular signature and clinical management. J Hepatol. (2013) 59:169–77. doi: 10.1016/j.jhep.2013.03.018 23542089

[71] Zignego AL Giannini C Gragnani L . HCV and lymphoproliferation. Clin Dev Immunol. (2012) 2012:980942. doi: 10.1155/2012/980942 22852020 PMC3407626

[72] Hermine O Lefrere F Bronowicki JP Mariette X Jondeau K Eclache-Saudreau V . Regression of splenic lymphoma with villous lymphocytes after treatment of hepatitis C virus infection. N Engl J Med. (2002) 347:89–94. doi: 10.1056/nejmoa013376 12110736

[73] Vallisa D Bernuzzi P Arcaini L Sacchi S Callea V Marasca R . Role of anti-hepatitis C virus (HCV) treatment in HCV-related, low-grade B-cell non-Hodgkin’s lymphoma: a multicenter Italian experience. J Clin Oncol. (2005) 23:468–73. doi: 10.1200/JCO.2005.06.008 15659492

[74] Arcaini L Vallisa D Rattotti S Ferretti VV Ferreri AJM Bernuzzi P . Antiviral treatment in patients with indolent B-cell lymphomas associated with HCV infection: a study of the Fondazione Italiana Linfomi. Ann Oncol. (2014) 25:1404–10. doi: 10.1093/annonc/mdu166 24799461

[75] Peveling-Oberhag J Arcaini L Bankov K Zeuzem S Herrmann E . The anti-lymphoma activity of antiviral therapy in HCV-associated B-cell non-Hodgkin lymphomas: a meta-analysis. J Viral Hepat. (2016) 23:536–44. doi: 10.1111/jvh.12518 26924533

[76] Carloni G Rinaldi M . Mechanisms and therapeutic strategies for HCV/HBV-associated B-cell non-Hodgkin’s lymphomas: a viewpoint. Oncol Res. (2026) 34:29. doi: 10.32604/or.2025.071847 41799526 PMC12963656

[77] Gao M . Antiviral activity and resistance of HCV NS5A replication complex inhibitors. Curr Opin Virol. (2013) 3:514–20. doi: 10.1016/j.coviro.2013.06.014 23896281

[78] Borges PHO Gras E Ferreira SB Paes Silva F Jr . Hepatitis C virus (HCV) proteases: structure, function and inhibition strategies. Enzymes. (2025) 58:151–82. doi: 10.1016/bs.enz.2025.06.003 41238296

[79] Lababidi JM Kabil MF Azzazy HME . Sofosbuvir: a comprehensive profile. Profiles Drug Subst Excip Relat Methodol. (2025) 50:1–41. doi: 10.1016/bs.podrm.2024.10.003 39855774

[80] Waheed Y Bhatti A Ashraf M . RNA dependent RNA polymerase of HCV: a potential target for the development of antiviral drugs. Infect Genet Evol. (2013) 14:247–57. doi: 10.1016/j.meegid.2012.12.004 23291407

[81] Merli M Rattotti S Spina M Re F Motta M Piazza F . Direct-acting antivirals as primary treatment for hepatitis C virus-associated indolent non-Hodgkin lymphomas: the BArT study of the Fondazione Italiana Linfomi. J Clin Oncol. (2022) 40:4060–70. doi: 10.1200/jco.22.00668 35714311 PMC9746784

[82] Tonutti A Polverini D De Nicola S Ceribelli A Soleri M De Santis M . The evolving scenario of HCV-related mixed cryoglobulinemia and B-cell lymphoma in the era of direct-acting antivirals. Expert Rev Anti Infect Ther. (2025) 23:19–30. doi: 10.1080/14787210.2024.2442475 39749733

[83] Arcaini L Besson C Frigeni M Fontaine H Goldaniga M Casato M . Interferon-free antiviral treatment in B-cell lymphoproliferative disorders associated with hepatitis C virus infection. Blood. (2016) 128:2527–32. doi: 10.1182/blood-2016-05-714667 27605512

[84] Thieblemont C Gomes Da Silva M Leppä S Lenz G Cottereau AS Fox C . Large B-cell lymphoma (LBCL): EHA Clinical Practice Guidelines for diagnosis, treatment, and follow-up. HemaSphere. (2025) 9:e70207. doi: 10.1002/hem3.70207 40995463 PMC12456099

[85] Gumà J Palazón-Carrión N Rueda-Domínguez A Sequero S Calvo V García-Arroyo R . SEOM-GOTEL clinical guidelines on diffuse large B-cell lymphoma (update 2025). Clin Transl Oncol. (2025) 27:4381–92. doi: 10.1007/s12094-025-04080-z 41129029 PMC12630300

[86] Hosry J Mahale P Turturro F Miranda RN Economides MP Granwehr BP . Antiviral therapy improves overall survival in hepatitis C virus-infected patients who develop diffuse large B-cell lymphoma. Int J Cancer. (2016) 139:2519–28. doi: 10.1002/ijc.30372 27501007 PMC5028297

[87] Cacoub P Comarmond C Vieira M Régnier P Saadoun D . HCV-related lymphoproliferative disorders in the direct-acting antiviral era: From mixed cryoglobulinaemia to B-cell lymphoma. J Hepatol. (2022) 76:174–85. doi: 10.1016/j.jhep.2021.09.023 34600000

[88] Terrier B Chaara W Dufat L Geri G Rosenzwajg M Musset L . Serum biomarker signature identifies patients with B-cell non-Hodgkin lymphoma associated with cryoglobulinemia vasculitis in chronic HCV infection. Autoimmun Rev. (2014) 13:319–26. doi: 10.1016/j.autrev.2013.11.001 24220267

[89] Basile U Marino M Gragnani L Napodano C Gulli F Pocino K . Sentinel biomarkers in HCV positive patients with mixed cryoglobulinemia. J Immunol Methods. (2020) 476:112687. doi: 10.1016/j.jim.2019.112687 31669506

[90] Fabris M Quartuccio L Sacco S De Marchi G Pozzato G Mazzaro C . B-lymphocyte stimulator (BLyS) up-regulation in mixed cryoglobulinaemia syndrome and hepatitis C virus infection. Rheumatol (Oxford). (2007) 46:37–43. doi: 10.1093/rheumatology/kel174 16735452

[91] De Re V Caggiari L De Zorzi M Repetto O Zignego AL Izzo F . Genetic diversity of the KIR/HLA system and susceptibility to hepatitis C virus-related diseases. PloS One. (2015) 10:e0117420. doi: 10.1371/journal.pone.0117420 25700262 PMC4336327

[92] De Re V De Zorzi M Caggiari L Lauletta G Tornesello ML Fognani E . HCV-related liver and lymphoproliferative diseases: association with polymorphisms of IL28B and TLR2. Oncotarget. (2016) 7:37487–97. doi: 10.18632/oncotarget.9303 27183918 PMC5122326

[93] Peveling-Oberhag J Crisman G Schmidt A Döring C Lucioni M Arcaini L . Dysregulation of global microRNA expression in splenic marginal zone lymphoma and influence of chronic hepatitis C virus infection. Leukemia. (2012) 26:1654–62. doi: 10.1038/leu.2012.29 22307176

[94] Gragnani L Fognani E Piluso A Zignego AL . Hepatitis C-associated B-cell non-Hodgkin lymphomas: the emerging role of miRNA-26b. J Hepatol. (2013) 59:1362–3. doi: 10.1016/j.jhep.2013.07.045 23978716

[95] Gragnani L Fognani E De Re V Libra M Garozzo A Caini P . Notch4 and MHC class II polymorphisms are associated with HCV-related benign and Malignant lymphoproliferative diseases. Oncotarget. (2017) 8:71528–35. doi: 10.18632/oncotarget.17655 29069725 PMC5641068

[96] Plummer M de Martel C Vignat J Ferlay J Bray F Franceschi S . Global burden of cancers attributable to infections in 2012: a synthetic analysis. Lancet Glob Health. (2016) 4:e609–16. doi: 10.1016/S2214-109X(16)30143-7 27470177

[97] Volesky-Avellaneda KD Morais S Walter SD O'Brien TR Hildesheim A Engels EA . Cancers attributable to infections in the US in 2017: A meta-analysis. JAMA Oncol. (2023) 9:1678–87. doi: 10.1001/jamaoncol.2023.4273 37856141 PMC10587828

[98] Schiller JT Lowy DR . An introduction to virus infections and human cancer. Recent Results Cancer Res. (2021) 217:1–11. doi: 10.1007/978-3-030-57362-1_1 33200359 PMC8336782

[99] de Martel C Georges D Bray F Ferlay J Clifford GM . Global burden of cancer attributable to infections in 2018: a worldwide incidence analysis. Lancet Glob Health. (2020) 8:e180–90. doi: 10.1016/S2214-109X(19)30488-7 31862245

[100] Manns MP Maasoumy B . Breakthroughs in hepatitis C research: from discovery to cure. Nat Rev Gastroenterol Hepatol. (2022) 19:533–50. doi: 10.1038/s41575-022-00608-8 35595834 PMC9122245

